# Sensory–Motor Loop Adaptation in Boolean Network Robots

**DOI:** 10.3390/s24113393

**Published:** 2024-05-24

**Authors:** Michele Braccini, Yuri Gardinazzi, Andrea Roli, Marco Villani

**Affiliations:** 1 Department of Computer Science and Engineering, University of Bologna, 47521 Cesena, Italy; andrea.roli@unibo.it; 2 Department of Mathematics, Informatics and Geosciences, University of Trieste, 34127 Trieste, Italy; yuri.gardinazzi@phd.units.it; 3 AREA Science Park, 34149 Trieste, Italy; 4 Department of Physics, Informatics and Mathematics, University of Modena and Reggio Emilia, 41125 Modena, Italy; marco.villani@unimore.it; 5 European Centre for Living Technology, 30123 Venice, Italy

**Keywords:** sensory-motor loop, Boolean networks, homeostasis

## Abstract

Recent technological advances have made it possible to produce tiny robots equipped with simple sensors and effectors. Micro-robots are particularly suitable for scenarios such as exploration of hostile environments, and emergency intervention, e.g., in areas subject to earthquakes or fires. A crucial desirable feature of such a robot is the capability of adapting to the specific environment in which it has to operate. Given the limited computational capabilities of a micro-robot, this property cannot be achieved by complicated software but it rather should come from the flexibility of simple control mechanisms, such as the sensory–motor loop. In this work, we explore the possibility of equipping simple robots controlled by Boolean networks with the capability of modulating their sensory–motor loop such that their behavior adapts to the incumbent environmental conditions. This study builds upon the cybernetic concept of homeostasis, which is the property of maintaining essential parameters inside vital ranges, and analyzes the performance of adaptive mechanisms intervening in the sensory–motor loop. In particular, we focus on the possibility of maneuvering the robot’s effectors such that both their connections to network nodes and environmental features can be adapted. As the actions the robot takes have a feedback effect to its sensors mediated by the environment, this mechanism makes it possible to tune the sensory–motor loop, which, in turn, determines the robot’s behavior. We study this general setting in simulation and assess to what extent this mechanism can sustain the homeostasis of the robot. Our results show that controllers made of random Boolean networks in critical and chaotic regimes can be tuned such that their homeostasis in different environments is kept. This outcome is a step towards the design and deployment of controllers for micro-robots able to adapt to different environments.

## 1. Introduction

The pace at which robot technology proceeds has rapidly stepped up in recent years. In addition to complex robots, such as humanoid ones and unmanned rovers, small robots of millimeter or micrometer size have been released. Such robots are often inspired by insects and bugs, and are usually equipped with a few simple sensors and actuators, and are characterized by limited computational capabilities. A prominent scenario for these robots is that of swarms to be applied in exploration of hostile environments and emergency intervention. The missions robots have to accomplish in these situations require capabilities such as autonomy and adaptiveness. AI techniques provide effective and efficient solutions to address these issues. Nevertheless, the reduced computational capabilities of micro-robots make these techniques often inapplicable, hence the need for alternative forms of control that are simpler yet still able to produce adaptive behaviors. A theoretical framework for defining this problem and finding solutions to it is that of embodied AI [1,2], which relies on the sensory–motor loop that connects the robot with the environment and makes it possible for a robot to adapt. The sensors produce the information the robot uses to determine its actions, which modify the environment through the effectors; this, in turn, generates possibly new sensor readings. Hence, the actions of the robot can influence its sensing. This feedback loop is at the basis of elementary yet non-trivial behaviors, such as gradient following and motion avoiding dangerous areas. The sensory–motor loop also plays a significant role in the integration in time of sensor signals, often making it possible to extract relevant information from the environment that is not directly perceived by sensors. For example, by combining movement and light perception, a robot can estimate the height of an object [3]. However, for a robot to be adaptive, mechanisms to change and tune the sensory–motor loop are needed. As beautifully stated by Ashby [4], organisms adapt to their environment “for the better”. In robotics, this condition is usually expressed in terms of a merit factor expressed in terms of a utility function, which guides the adaptation process. The utility function is mostly based on the specific task the robot has to accomplish and it is given by the designer beforehand. This is of course a common and feasible approach when the most relevant features of the environment and the task can be modeled in advance. Nevertheless, in some situations, like emergencies, such predefined utility functions might not capture the actual “good” for the robot. In such cases, the robots should be equipped with general, task-agnostic rules for acting and adapting. These rules are commonly called values. The ultimate value is self-maintaining, which can be formally stated as homeostasis, i.e., the capability of keeping the system in a working condition despite the changes in the environment. Notably, the ability of maintaining homeostasis in changing environments can be interpreted as a way of making sense of the environmental features that are relevant to the robot. This perspective is not new, as it has been thoroughly discussed in fields such as cybernetics and cognitive systems [3,4,5,6,7,8,9,10,11,12,13]. Keeping this context as a reference, in this work, we discuss the results of a study aimed at investigating and exploring possible adaptive mechanisms for micro-robots controlled by simple circuits. Since the typologies and features of micro-robots are—and will be—extremely varied, we focus on a kind of control software that can match any specific instance of physical robots. Therefore, the level of abstraction of our investigation is at the control layer, independently of the actual physical robot. In particular, we concentrate our attention to control software based on Boolean networks and we study the properties of simulated robots that have to keep their homeostasis in front of environment changes. This work is aimed at providing guidelines and suggestions for designing new control mechanisms capable of enabling adaptivity, especially in micro-robots. Other authors have approached the topic of homeostasis from different perspectives, e.g., through the lens of active inference [14] and within the framework of active perception [15], to name just a few.

Our investigation is inspired by cybernetics, minimally cognitive robotics and dynamical systems, and tries to bridge this theoretical background to robot control methods and technologies. To the best of our knowledge, this is the first time such a study is presented. Nevertheless, Boolean network controllers for robots—and their connections to dynamical criticality—have been already studied, mainly by some authors of this contribution, [16,17]. In [17], an offline adaptation of a robot controlled by Boolean networks has been presented, while online adaptation has been investigated in [16], where it is shown that Boolean networks poised at a dynamical regime between order and disorder are the one that enable the robot to achieve the best performance. The adaptive mechanisms devised in [16] are similar to those used in this work. Nevertheless, in previous works, robots learned to accomplish a specific task in a given environment, while they have to be able to adapt and maintain their operative status in this work.

In Section 1, we introduce the model used in our experiments. As we are dealing with simulated robots, we will use the terms agent and robot interchangeably. In Section 2, we present the robot’s and environment’s models along with the adaptive mechanism employed by the robot to achieve homeostatic condition when coping with a changed environment. The results for the specific scenario of coupled robot–environment systems studied are described in Section 3, while Section 4 discusses their possible general implications. Section 5 is devoted to the presentation of the conclusion.

## 2. The Model

The main elements that have to be defined for setting up experiments of autonomous agents in a changing environment are (i) an autonomous agent, and (ii) a dynamic environment in which the moves of an agent can have effects in a simple (enough to be useful for designing experiments) but effective way. Further, (iii) it is necessary to allow the agent to establish what is advantageous and what is not.

This last characteristic is perhaps the most delicate, as we aim at meanings/benefits to emerge without design, or to be “autonomously decided” by the system immersed in a dynamically changing environment, without an explicit design by the experimenters. At the same time, much of the meaning/usefulness actually depends on the interaction between the agent and the particular environment and on the active feedback loops. The role of the sensory–motor mechanisms lies precisely in this last aspect, which we will focus on in this work. In order to highlight this last aspect, we will assume that the agent is already in a “good” situation due to a previous history (evolution for living beings, explicit design for artificial systems), and it is exposed to new environments. A general goal of any agent in such a situation is to keep its internal situation stable and coherent (homeostasis). If the new environment has changed this situation, the agent should react by influencing the environment in order to restore the state of well-being.

### 2.1. Agent’s Model

We are interested in simulating an agent moving in an unknown environment: the agent and the environment each have their own dynamics, while the exchange of information between the two systems occurs through the sensors and actuators of the agent. The internal states of the agent and their relationships are represented through a random Boolean network (RBN). The environment must have non-obvious dynamics, which can be modified through the interaction of an external system (the robot) with the features of the environment itself. An elegant way to model a reactive environment is to realize it via another RBN.

Boolean networks (BNs) are simple but powerful discrete models of gene regulatory networks, introduced by Kauffman [18,19]. BNs can be defined mathematically as a directed graph with N nodes whose node states are described by Boolean variables $x_{i}\;,\;i=1,\;...\;,N$, which can take the values ON or OFF, and their evolution over time is described by Boolean functions $f_{i}\;(x_{i_{1}},\;...\;,\;x_{i_{Mi}})$, where Mi is the number of inputs of node i. Random Boolean networks (RBNs) are a special class of BNs, a class determined by parameters that impose constraints on the connections between nodes and on their dynamics.

A noteworthy RBN class is one in which each node receives an exact number of inputs, determined by the K parameter, chosen randomly from the other nodes and avoiding self-loops, and in which the probability of having the value 1 in each entry in the truth tables of the Boolean functions is determined by the p parameters, called bias. In these works [20,21], the authors showed that this mathematical relationship between p and K, $K=[2p\;(1-p)]{\;}^{-1},$ describes the set of RBNs that fall in the order-disorder phase transition, the so-called critical line for RBNs. Perturbations in RBNs in the ordered dynamical regime generally die out quickly, whereas they spread out in disordered networks. Networks in disorder regime are also characterized by very long cyclic attractors.

In contrast, networks in the critical regime—those falling into the region described by the above equation—show a balance between adaptability and robustness that has led to the formulation of the conjecture that life and computation exist at the edge of chaos, the criticality hypothesis [22,23,24,25].

Critical RBNs have proven capable of properly reproducing many biological processes [26,27,28,29,30,31,32,33,34] and have proven efficient when used as controllers of artificial agents, such as robots [27].

**Figure 1:**
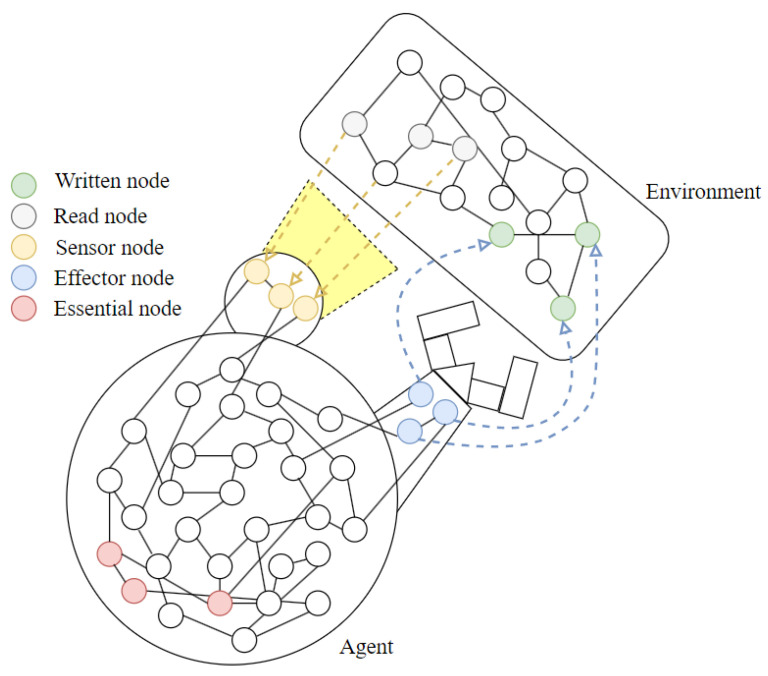
Agent interacting with an environment. It detects the environment features by using its sensors and acts on some features of the environment by using the effector nodes. The essential nodes are highlighted in pink.

The robot’s effectors can therefore be represented through nodes that are able to influence the state of nodes belonging to the environment (hereinafter called “features”), while its sensors are nodes that receive signals from some features of the environment. For simplicity, we assume that the value of a sensor node is influenced by only one feature of the environment, and similarly the value of an effector node influences only one feature of the environment. In a physical implementation, this implies that the Boolean value of each output node of the network controls an effector via a given encoding (e.g., in the case of a device controlled by an impulse, the Boolean values naturally correspond to minimum/maximum voltage applied). Similarly, an input node assumes a Boolean value which is the result of an encoding from a possibly continuous to Boolean domain (e.g., values from [0, 1] can be converted simply by introducing a threshold). The state of the robot’s well-being is measured by the distance from homeostasis of a subset of its nodes (the “essential nodes”). To avoid trivial dynamics, the three sets (sensor nodes, effector nodes and essential nodes), whose sum of cardinalities is less than the total number of robot nodes, are non-overlapping (see Figure 1).

There is a “universal time”, common to the robot and the environment, which proceeds in discrete steps and regulates the succession of events. However, the internal processes of the robot can be slower or faster than the environmental processes: each of the two systems therefore has an internal variable (called, respectively, agent_step and env_step) which establishes how many “universal” steps must pass to allow the application of the internal dynamical rules. If the values of these variables are the same both systems proceed in synchronization, otherwise the system that has a higher value is “slower” than the other one; as a special case, if the value of both the variables is equal to 1, robot and environment proceed paired, at maximum speed.

It is therefore needed to describe the interaction between two systems that can have different speeds. For each sensor node, it is necessary to define (i) the perceived value of the environmental feature to which it is associated and (ii) its response to that value. It was therefore decided that (a) the perceived value is equal to the average of a number of universal steps of the environment equal to agent_step, while (b) the response of the sensor node is equal to “0” or “1” depending on that the perceived average is below or above a threshold *θ*∈]0,1[ (in the particular case that *env_step* is equal to 1, the perceived value is the value of the node itself, and any threshold does not modify its value).

**Figure 2:**
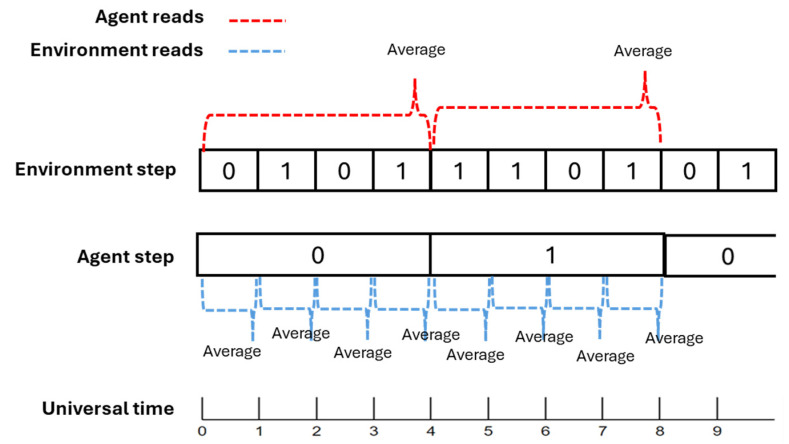
Example of how nodes are read with an environment with env_step = 1 and agent with agent_step = 4. In this case, the environment just averages on one value of the agent, while the agent averages the 4 last environment steps.

An effector node acts on a specific feature of the environment. The mechanism is symmetrical to the previous one: (a) the value perceived by the environmental feature is equal to the average of a number of universal steps of the robot equal to env_step, while (b) the response of the feature is equal to “0” or “1” depending on that the perceived average is below or above a threshold *θ*∈]0,1[. This “response” is the value that the node effector of the robot manages to apply on the feature of the environment. See Figure 2.

As anticipated, we assume that the robot without an environment is already in its ideal situation: once exposed to an unknown environment it must therefore act in such a way as to restore this situation, at least for the essential nodes. The ideal profile is therefore defined as the average, essential node by essential node, taken over a *η* steps performed by the robot without any environment (be ${\underline{X}}_{id,\eta }$ the vector of these averages).

We then evaluate the distance from this ideal situation during the life of an agent T times (each evaluation being interspersed with ω agent steps) and consider the average of these values; in this paper we assume the Euclidean distance:

(1) $$D=\frac{\sum_{n=1}^{T}d({\underline{X}}_{\mathrm{e},\omega },{\underline{X}}_{id,\eta })}{T}=\frac{\sum_{n=1}^{T}{\left\|{\underline{X}}_{\mathrm{e},\omega },{\underline{X}}_{id,\eta }\right\|}_{2}}{T}$$

The lifetime of an agent measured in universal steps is therefore equal to T “trials”, multiplied by ω steps, multiplied by *agent_step*:

(2) $$L_{agent}=\omega \cdot T\cdot agent\_step$$

### 2.2. Adaptive Mechanisms

To use the characteristics of the environment in order to maintain its well-being (homeostasis), the robot must therefore choose the features on which to act and those from which to derive utility.

To avoid trivial situations in this paper we do not allow the robot to choose the features of the environment from which to draw utility, nor its nodes that are connected to these features. The aim of the robot is therefore given its characteristics (its internal structure and the characteristics of the sensor nodes), to discover where and how to act on the environment (through the choice of the effector nodes and the features of the environment on which they act), in order to receive from it (through the features of the environment to which the sensor nodes are connected) the stimuli needed to maintain homeostasis (minimize the distance of the essential nodes from their ideal profiles).

We can think of the robot’s learning as a succession of its versions, each deriving from the best of the previous ones. Each version has the same lifespan and starts from the same initial conditions and is evaluated in the way just described (Equation (1)). The robot thus tests in an organized manner the various possibilities offered by the interaction between itself and the environment, until it reaches the maximum possible number of tests. In each change step, the next version is accepted only if its distance from the ideal profiles of the essential nodes is equal to or lower than that of the previous one: at the end of the process the final version of the robot is obtained.

Changes can be performed following three strategies:

1. Change the effector nodes, leaving the features of the environment on which they act unchanged;
2. Maintain the effector nodes, and change the features of the environment on which they act;
3. Change both the effector nodes and the features of the environment on which they act.

**Figure 3:**
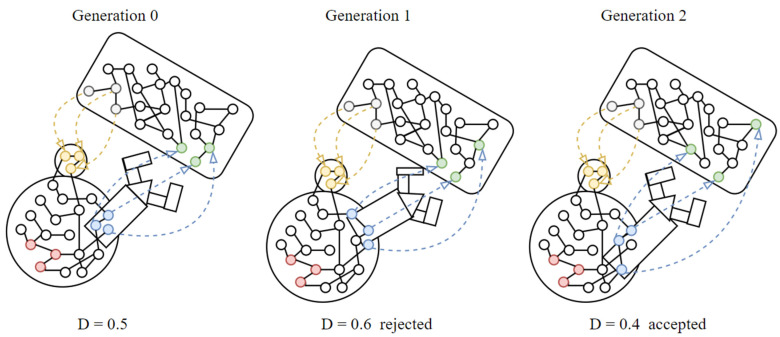
Example of evolution of an agent. The generation 1 is rejected because a higher value of D.

The basic steps defining adaptation strategy 1 and 3 are actually the same as those used in the online adaptation mechanism proposed in [27]. See Figure 3.

## 3. Results

Below, we will present results for the case that, roughly speaking, we can describe as “fast environment and slow robot”, that is, the case where *env_step* = 1 and *agent_step* = 10.

In general, this scenario should give the dynamics of the environment time to relax on one of its attractors upon the perturbation received by the agent on its features.

This should, in turn, allow the robot to receive a response (sensory input) mediated by the environment that is as time consistent as possible with the action that triggered it (performed by the robot itself), thus unaffected by possible fluctuations resulting from transients of the environment dynamics.

We are also interested in observing how the dynamical regime expressed by a Boolean network can influence its bouquet of responses if immersed in unknown environments.

We calculate Derrida’s coefficient to evaluate the dynamical regime in which a Boolean network operates. Derrida’s coefficient *λ* measures the average level of propagation of a perturbation after a simulation step. Statistically, *λ* > 1 characterizes chaotic networks, *λ* < 1 ordered networks while *λ* = 1 critical networks. To calculate it for a single Boolean state, we copied or negated one of its variables; we then performed a synchronous update for both the original and the perturbed state and finally measured the Hamming distance between the two resulting states. Next, we averaged the values by repeating this procedure 80 times for each of the 250 initial conditions, resulting in 20,000 total occurrences.

### 3.1. The Three Strategies

We carried out a series of experiments, in which robots were immersed in unknown environments. These environments must have their own dynamics and timing: for this purpose we once again used the RBN framework. We, therefore, created 10 instances of RBNs, each having 50 nodes and average connectivity equal to 3: the bias *p* equal to 0.21 implies a critical dynamical regime (as a check, we measured the Derrida coefficients of each environment, which all turned out to be close to 1.0).

In a preliminary series of experiments, we evaluated the homeostasis recovery capacity of ensembles of robots (with critical and chaotic dynamical regimes) once they were exposed to each of 10 environments. Once immersed in an environment, each robot was typically distanced from its “ideal” state, and applied one of the three strategies reported above to decrease (if not eliminate completely) this distance (measured as in Equation (1)). In each environment *E_j_*, we used the relative decrease in the distance from the ideal profile as an evaluation of the capacity for homeostasis of robot *R_i_*:

(3) $$G_{i}(E_{j})=\frac{D_{initial}^{i}(E_{j})-D_{final}^{i}(E_{j})}{D_{initial}^{i}(E_{j})}$$

so that the performance of each robot R_i_ is the average relative decrement:

(4) $$P_{i}=\frac{1}{10}\sum_{j=1}^{10}G_{i}(E_{j})$$

In the following, we will use robots having an internal system schematized by using RBNs composed of 100 nodes, of which 3 sensor nodes, 3 effector nodes and 3 effective nodes. The three subsets are non-overlapping, and direct connections (i) between sensor nodes and effector nodes, (ii) between sensor nodes and effective nodes, and (iii) between effective nodes and effector nodes are excluded.

Statistics using ensembles of 50 robots have indicated that the third strategy is the one that allows for better versatility (see Table 1): in what follows we will therefore focus on this modality.

In these experiments, as in the following ones, the difference between the robots of the ordered, critical and chaotic sets lies—without loss of generality—in the Boolean functions used. Each created topology was then combined with sets of Boolean functions having biases equal to 0.1, 0.21 and 0.5: the common connectivity *k =* 3 leads these RBNs, if isolated, to exhibit, respectively, ordered, critical or chaotic behaviors.

The ordered ensembles have always shown zero average performance values: we will therefore avoid dealing with them. In Table 1, the data seem to indicate a better performance than chaotic RBNs: to interpret the result, however, a more detailed analysis is necessary (see Section 3.3). During the research, we tested different sets of parameters: in the simulations presented in this work, we used the values indicated in Table 2.

### 3.2. A Common Behavior

**Figure 4:**
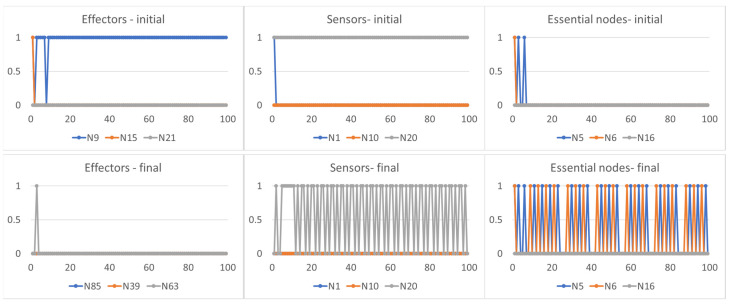
The first row shows the trajectory of the effector, sensor and essential nodes of the critical agent C1 when it is exposed to one of the environments. The second row reports the trajectory of the effector nodes (in the case the agent has modified its effector set, different from the previous ones), sensor and essential nodes of the same agent once it has adapted to the new environment. The features of the environment on which the agent exercises its final action can be different from those on which it initially acted. It is possible to note that a constant action on the environment (first plot of the second row) leads to an oscillating signal in the sensor nodes (second plot of the second row): this action brought the average activity of the essential nodes (third plot of the second row) close to the homeostatic average, in this case equal to [0.2, 0.2, 0.0].

**Figure 5:**
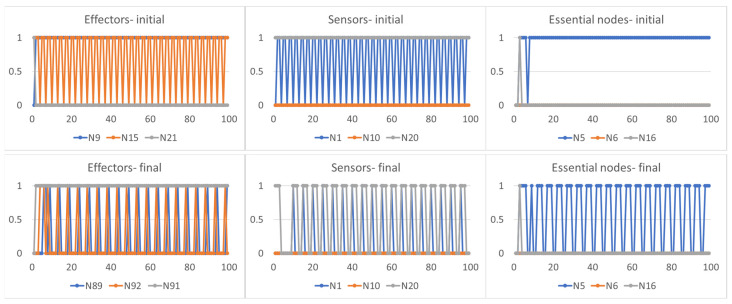
The first row shows the trajectory of the effector, sensor and essential nodes of the critical agent C2 when it is exposed to one of the environments. As in Figure 4, the second row reports the trajectory of the effector, sensor and essential nodes of the same agent once it has adapted to the new environment. It is possible to note that a different oscillating action on the environment (second row, first plot) leads to an oscillating signal in the sensor nodes (second row, second plot) able to induce on the essential nodes (second row, third plot) an average activity the close to the homeostatic average [0.2, 0.0, 0.8].

**Figure 6:**
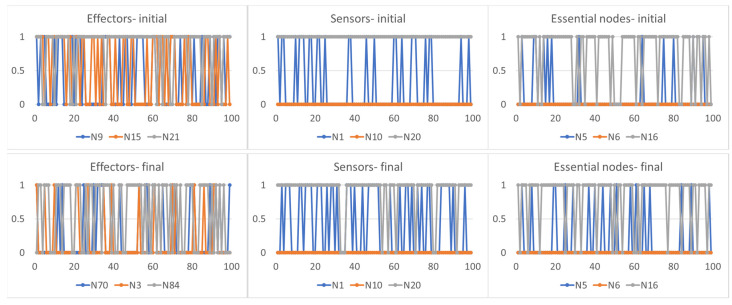
The first row shows the trajectory of the effector, sensor and essential nodes of the dis-ordered agent D1 when it is exposed to one of the environments. As in Figure 4, the second row reports the trajectory of the effector, sensor and essential nodes of the same agent once it has adapted to the new environment. It is possible to note that a different oscillating action on the environment (second row, first plot) leads to an oscillating signal in the sensor nodes (second row, second plot) able to induce on the essential nodes (second row, third plot) an average activity the close to the homeostatic average [0.6, 0.0, 0.1].

An interesting characteristic, common to all adaptation strategies, concerns the type of action that agents can exercise once adapted to the new environment. Agents typically change both the features of the environment influenced by them (when they can, and therefore with the exception of strategy (1) and their effector nodes: the action conducted by these nodes does not, however, always have the same effects. In fact, a constant action exercised on particular areas of the environment may have the effect of an oscillating signal on the sensor nodes (the critical agent C1 in Figure 4), while an oscillating action on other areas of the environment causes a different signal oscillating on the sensor nodes (the critical agent C2 in Figure 5 or the disordered agent D1 in Figure 6). In these cases, the need for a final oscillating situation of the essential nodes depends on the fact that the three agents all require average node activities different from stable values equal to 0 or 1, but the notable point is that this situation can be obtained either by exercising a constant action and an oscillating one.

The agents therefore used the environment to which they were exposed in a non-trivial way, including its dynamic characteristics in their effector–sensor loop to achieve homeostasis. Remarkably, the methods used are such as to make this action difficult to identify based on internal observations alone: a similar action (the induction of particular oscillations in the essential nodes) was achieved in one case through a constant action on the environment (agent C1) and in other cases through an oscillating action (agents C2 and D1).

The scheme we adopted therefore allows us to identify the dynamic role of the coupling between the environment and the agent, highlighting the explicit action of the agent of incorporation into the effector–sensor loop of the dynamics of the environment for the purposes of its own well-being.

### 3.3. The Third Strategy

We therefore focused on the third strategy, using larger ensembles (each ensemble being composed of 500 RBNs). It is possible to make some interesting considerations.

**Figure 7:**
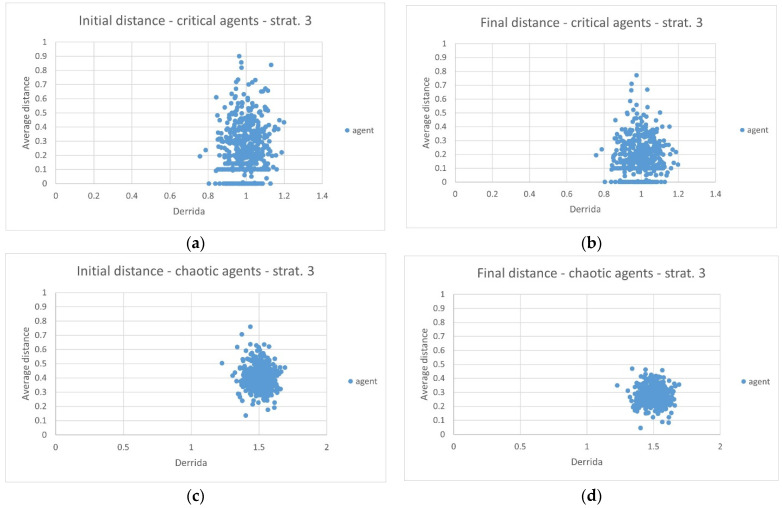
(**a**) The initial distance from the ideal profile of the 500 critical RBNs as soon as they are exposed in each of the 10 environments (each point corresponds to the average distance value over the 10 environments). (**b**) The average distance from the ideal profile of the 500 critical RBNs once the adaptation has been performed following strategy 3. (**c**) The average initial distance from the ideal profile of the 500 chaotic RBNs. (**d**) The average final distance from the ideal profile of the 500 chaotic RBNs once the adaptation has been performed following strategy 3. In all graphs, the X axis indicates the Derrida coefficient of each individual RBN.

A first observation concerns the fact that the critical ensemble shows a great variety of behaviors, from RBNs that have not received any perturbation from the environment, to RBNs that have been strongly perturbed, in a way that is little influenced by the Derrida coefficient, consistently always placed around the value 1.0 (Figure 7a). The chaotic ensemble shows much greater uniformity (Figure 7c).

Both ensembles show improvements once the RBNs are allowed to adapt to different environments (Figure 7b,d). Again, the RBNs belonging to the chaotic ensemble show a uniformity of behavior that seems absent from the critical ensemble.

This diversity of behavior allows us to make various considerations. The chaotic regime (better to say “extremely disordered”) leads to a uniformity of behavior, little dependent on the details of the individual instances. All RBNs are perturbed by external environments, without exception; all RBNs show traces of improvement once adaptation is allowed. The RBNs belonging to the critical ensemble show extreme diversity: many are not perturbed by the environments to which they have been exposed, many have been highly perturbed, more than the most perturbed chaotic RBN. Many of the perturbed RBNs show improvements. From an evolutionary point of view, this greater diversity of behavior is an advantage, allowing the subsequent selection of the variant most suited to the need. Table 3 shows how many of the critical RBNs were not perturbed by the new environments, and how many, even if initially perturbed, did not improve.

It is interesting to observe what happens to critical RBNs that have been perturbed by exposure to unknown environments and improved once they were allowed to adapt (a situation similar to that of the RBNs belonging to the chaotic ensemble).

**Figure 8:**
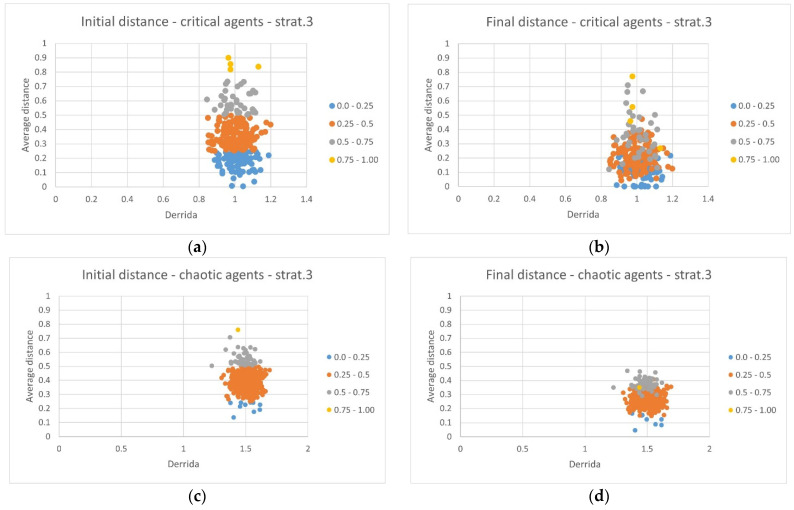
(**a**) The average initial distance from the ideal profile of the critical RBNs that were perturbed and then improved through the application of strategy 3, highlighted in different colors depending on the initial distance. (**b**) The average distance from the ideal profile once the adaptation has been performed following strategy 3. (**c**) The average initial distance from the ideal profile of the chaotic RBNs. (**d**) The average final distance from the ideal profile of the chaotic RBNs once the adaptation has been performed following strategy 3. In all graphs, the colors identify 4 bands depending on the distance: blue for the interval [0.0–0.25[, orange for the interval [0.25–0.50[, gray for [0.50–0.75[ and yellow for [0.75–1.00].

In this case, it is possible to see that there are critical RBNs capable of completely zeroing out the distance from their ideal profiles, and that there are RBNs that, despite starting from a very disadvantaged position, are able to express notable recoveries (the blurring of the colors in Figure 8b, in which the yellow dots and gray dots show even greater improvements than orange and blue dots).

Similar capabilities are not shown by the RBNs belonging to the chaotic ensemble, which maintain uniformity of behavior (the stable division into separate colored bands). Furthermore, in this situation, the RBNs belonging to the critical ensemble also show an average recovery capacity higher than that of the RBNs belonging to the chaotic ensemble (Table 4).

In other words, if we extract from the critical ensemble the RBNs that are sensitive to the environment and capable of improvement, we obtain systems that are typically better than the corresponding systems showing a chaotic dynamical regime. The results therefore agree with Kauffman’s hypotheses, which imagines a particular role for critical dynamical regimes, which (i) show greater initial variability are susceptible to selection and (ii) are capable of supporting a high degree of adaptability. On the other hand, the uniformity of behavior of the chaotic ensemble prevents the possibility of having particularly high-performance RBNs.

## 4. Discussion

The model chosen for our experiments, although simple, makes it possible to address fundamental questions concerning robot adaptation. Rephrasing Ashby [4] in current terms, we have explored possible ways for achieving adaptation (“identify the nature of the change which shows learning”) and investigated the circumstances that make this adaptation more efficient (“to find why such changes should tend to cause better adaptation for the whole organism”).

The use of RBNs to control the agents on the one hand provides generality to the results we observed—indeed, only the dynamical regime matters—and, on the other hand, it shows that network-based controllers can be subject to adaptive mechanisms that confer non-trivial adaptive capabilities to robots. Furthermore, the choice of homeostasis as a merit factor for guiding adaptation addresses the issue of providing robots with autonomy: once a robot is capable of changing some of its control parameters such that its survival is preserved, then other task-specific utility functions can be optimized. This capability may well constitute a basic competence for a layer in a subsumption architecture [35].

A prudent reader might observe that a limitation of our results is that they have been attained in simulation only. Tests on real robots are indeed required to thoroughly assess the robustness of the phenomena we observed. However, previous works with Boolean network controlled robots [17] have validated on real robots (i.e., e-puck robots [36]) the results obtained in simulation. Furthermore, the adaptive process that we used is analogous to an adaptive walk in a configuration space, which can be considered as a special case of evolutionary algorithms used in robotics. Many results in evolutionary robotics and automatic design of control software for robots have been validated on real robots, for example [37,38,39,40,41,42]. Therefore, we are rather confident that the core behavior observed can be also observed when the controller we have studied is implemented on physical robots.

Central in our model is the adaptation of the sensory–motor loop, which is at the basis of behavior-based robotics. Here, we suppose that the sensors of the robot are given and not subject to adjustments, whereas the actuators are those devices that can be adapted to the environment in such a way that the robot can keep its homeostasis. As a side comment, we observe that this setting loosely resembles approaches based on the free energy principle [43], in which actions are taken such that the free energy of the system is minimized. Having only the actuators that can undergo changes clearly shows whether the agent is actually able to choose suitable actions to adapt: when the agent is placed in a new environment and its homeostasis is negatively perturbed, only by means of proper actions it can influence the environment to produce good and valuable inputs to its sensors. This has two main implications. The first one is that robots capable of adapting successfully exploit the features of the new environment to which they are exposed. In other terms, the anomalies produced by the environment trigger new effective behaviors in the robots. Therefore, effective behavior control in robots can profit from the combination of some degree of adaptation with tests in differing environments. The second important consequence is that the new action-sensor feedback, created by adaptation, that restores homeostasis in the agent identifies a sense-making property: the agent selects those features of the environment that matters to its survival and learns how to act on them under the conditions posited by the sensors and the internal mechanisms of the agent.

Finally, our results provide support to the conjecture that critical regimes are the most suited for adaptation and evolution. Another proof of the advantage of critical networks as controllers in Boolean network robots was already observed in the work [16]. The cited work analyzed the performance of an online adaptation scheme very similar to the one used in this paper, but employing real robots in obstacle avoidance and foraging tasks. However, the results reported in this work are more general, in that the Boolean network representing the agent does not attempt to improve a task-specific figure of merit, but instead tries to adapt to achieve its homeostatic condition.

This result can be generalized to other network-based controllers, such as nanowire networks [44].

## 5. Conclusions

In this work, we have investigated how robots controlled by Boolean networks can adjust their sensory–motor loop to adapt to environment changes, such that their homeostasis is restored. Some general implications of the results we obtained have been discussed in the previous section. However, we would like to point out that this work, through the proposed conceptual and experimental framework, paves the way for the discovery of the general principles that enable agent homeostasis in scenarios characterized by changing environments. The importance of the criticality of the agent’s controller adds to Ashby’s “Principle of Requisite Variety” [45,46] and begins to outline the framework of a truly homeostatic robot, a crucial first step toward robot autonomy and possibly to machines endowed with feeling [47]. A possible roadmap of future work involves testing this agent–environment framework (i) with real robots endowed with a physical body (ii) with the ability to adapt both its robotic morphology and its controller; (iii) with changing environments (ecological niches); (iv) in coevolutionary settings (i.e., both the agent and environment can change in response of the other previous and current perceivable actions); and finally (v) in multi-robot scenarios. In addition, our efforts will be devoted to characterize the processing of information flow that homeostasis induces between sensors, controllers, actuators, and the environment, which can be analyzed with Information Theory measures.

We are currently running experiments with larger networks to assess the results concerning the dynamical regimes. In addition, experiments with different relative update frequencies between agents and environment are under study. In addition to these further analyses, feasibility studies concerning the implementation on physical robots of the mechanisms we have studied in simulation are planned. Micro-robots can now be cheaply produced and it is plausible that they can be equipped with network-based controllers. In these robots, the essential variables might be represented by energy and efficiency parameters; therefore, basic capabilities of keeping these variables within working ranges would be of extreme utility. This survival competence can then provide the basis for more advanced and special purpose behaviors. Finally, the experimental setting we have designed makes it possible to investigate the relation between adaptation and high-level principles, such as the free energy principle and autopoiesis.

## Figures and Tables

**Table 1:** The average performances of ensembles (whose cardinality is equal to 40) of critical and chaotic robots when using the three adaptation strategies. The average is made on the relative decrease (Formula (3)). Strategy 3, which allows changes both in the choice of effector nodes and in the environmental features touched, has better results than the other two strategies.

	Average Performance		
	Ordered RBNs	Critical RBNs	Chaotic RBNs
STRAT. 1	0	0.075	0.196
STRAT. 2	0	0.131	0.264
STRAT. 3	0	0.194	0.308

**Table 2:** Values of the main parameters used during the simulations in this work. The length of each simulation is equal to omega*trial_number*trial_step *=* 280,000 “universal steps”. The probability that an effector node is modified (prob_change_effector) or of modifying the choice of a feature of the environment to be influenced (prob_change_feature) is equal to 0.0 or 0.5 depending on the strategy chosen; the sensor nodes and the environmental features they monitor do not change.

Variable	Value	Variable	Value
trial_number	2800	Number of nodes (agent)	100
trial_step	20	Number of nodes (environment)	50
*w*	5	Average connectivity k	3
prob_change_sensor	0;	Bias: Ordered ensemble	0.1
prob_change_effector	0; 0.5	Bias: Critical ensemble	0.21
prob_change_feature	0; 0.5	Bias: Disordered ensemble	0.5

**Table 3:** The table shows in the first row the number of critical RBNs that have been improved by adaptation phase, i.e., whose final distance from the homeostatic condition is less than the initial distance. The second row shows the networks that have been affected by the change in environment, but have not been able to move closer to their homeostatic condition, i.e., for the network *i* it occurs that $D_{final}^{i}(E_{j})\;\geq \;D_{initial}^{i}(E_{j})$. Finally, the last row shows the networks whose homeostasis has not been perturbed by environmental change.

# RBNs	Critical RBNs
Improved	266
Did not improve but perturbed	173
Not perturbed	61

**Table 4:** The average performances of the ensembles of critical and chaotic robots (whose cardinality is 500 for chaotic RBNs and 266 for critical RBNs that showed improvement) when using the third adaptation strategy, i.e., the best performing one (see Table 1).

	Critical RBNs (Only the Improved Ones)	Chaotic RBNs
MIN	0.001	0.115
MAX	1.000	0.658
AVERAGE	0.382	0.310
MEDIAN	0.377	0.300

## Data Availability

Data are contained within the article.
